# Comparison of Inhibitor and Substrate Selectivity between Rodent and Human Vascular Adhesion Protein-1

**DOI:** 10.1155/2020/3270513

**Published:** 2020-01-20

**Authors:** Ryo Kubota, Michael J. Reid, Kuo Lee Lieu, Mark Orme, Christine Diamond, Niklas Tulberg, Susan H. Henry

**Affiliations:** Acucela Inc., 818 Stewart St., Suite 1110, Seattle, WA 98101, USA

## Abstract

Vascular adhesion protein-1 (VAP-1) is an ectoenzyme that functions as a copper-containing amine oxidase and is involved in leukocyte adhesion at sites of inflammation. Inhibition of VAP-1 oxidative deamination has become an attractive target for anti-inflammatory therapy with demonstrated efficacy in rodent models of inflammation. A previous comparison of purified recombinant VAP-1 from mouse, rat, monkey, and human gene sequences predicted that rodent VAP-1 would have higher affinity for smaller hydrophilic substrates/inhibitors because of its narrower and more hydrophilic active site channel. An optimized *in vitro* oxidative deamination fluorescence assay with benzylamine (BA) was used to compare inhibition of five known inhibitors in recombinant mouse, rat, and human VAP-1. Human VAP-1 was more sensitive compared to rat or mouse VAP-1 (lowest IC_50_ concentration) to semicarbazide but was least sensitive to hydralazine and LJP-1207. Hydralazine had a lower IC_50_ in rats compared to humans, although not significant. However, the IC_50_ of hydralazine was significantly higher in the rat compared to mouse VAP-1. The larger hydrophobic compounds from Astellas (compound 35c) and Boehringer Ingelheim (PXS-4728A) were hypothesized to have higher binding affinity for human VAP-1 compared to rodent VAP-1 since the channel in human VAP-1 is larger and more hydrophobic than that in rodent VAP-1. Although the sensitivity of these two inhibitors was the lowest in the mouse enzyme, we found no significant differences between mouse, rat, and human VAP-1. Michaelis-Menten kinetics of the small primary amines phenylethylamine and tyramine were also compared to the common marker substrate BA demonstrating that BA had the highest affinity among the substrates. Rat VAP-1 had the highest affinity for all three substrates and mouse VAP-1 had intermediate affinity for BA and phenylethylamine, but tyramine was not a substrate for mouse VAP-1 under these assay conditions. These results suggest that comparing oxidative deamination in mouse and rat VAP-1 may be important if using these species for preclinical efficacy models.

## 1. Introduction

Vascular adhesion protein-1 (VAP-1) is involved in leukocyte adhesion at sites of inflammation [1] and is predominantly expressed in vascular endothelium, smooth muscles, and adipocytes as a membrane-bound ectoenzyme [2]. Biochemical functional assays demonstrated that VAP-1 had enzymatic activity as an amine oxidase that was requisite for adhesion [3]. Oxidative deamination activity by this copper-containing enzyme was distinguished from flavin adenine dinucleotide (FAD) cofactor-containing monoamine (MAO) and polyamine oxidases by its tissue distribution, subcellular location, and selective inhibition by semicarbazide [4]. Through sequence analysis, it was discovered that the VAP-1 is identical to the primary amine oxidase, semicarbazide-sensitive amine oxidase (SSAO) [3]. VAP-1 is a member of the copper-containing amine oxidases (CAOs) that are found in many organisms and have similar properties across most mammalian species [5]. Similar to other CAOs, VAP-1 was shown to have a unique quinone cofactor, topaquinone (TPQ; [6]), generated by posttranslational modification of a tyrosine in the active site [7] which participates in the oxidative deamination of primary amines, consuming oxygen, in the production of an aldehyde, hydrogen peroxide, and ammonia [8, 9]. Aside from benzylamine (BA) being a good substrate for CAOs and MAOs, various biogenic amines are substrates of VAP-1 to varying degrees in different species and tissue/plasma sources *in vitro* including tyramine (TYR), *β*-phenylethylamine (PEA), and tryptamine [8, 9], as well as methylamine and aminoacetone [8, 9], yet the physiological significance of these substrates remains uncertain.

Increases in VAP-1 activity and/or protein levels have been observed in many inflammation-associated diseases [10], such as primary sclerosing cholangitis [11], chronic obstructive pulmonary disease [12], atherosclerosis [13], and chronic liver disease [14]. Inhibition of the amine oxidase activity of VAP-1 has been shown to abrogate the recruitment of leukocytes, especially neutrophils, to sites of inflammation *in vivo* and the transendothelial migration of leukocytes *in vitro* [15, 16]. These findings have led to medicinal chemistry efforts to inhibit VAP-1 deamination activity as an approach to anti-inflammation therapies [17–19].

Effective inhibition of VAP-1 in experimental animal models of inflammation has been reviewed, yet poor cross-species selectivity has complicated the development of this effort [18, 20]. Various hydrazine compounds have been investigated as inhibitors of VAP-1 in bovine lung microsomes where hydralazine (HYD) was twenty times more potent than semicarbazide (SEM) when conincubated with benzylamine (BA) while phenylhydrazine and phenelzine were over 100 times more potent than semicarbazide [21]. The novel hydrazine, LJP-1207, was shown to be a potent inhibitor of recombinant human VAP-1 (rhVAP-1) as well as in rat lung and human umbilical cord homogenates. LJP-1207 was effective in reducing inflammation after oral administration to mice in models of ulcerative colitis and LPS-induced endotoxemia and rats in a carrageenan footpad model of inflammation [22, 23]. The novel guanidine linked to a thiazole synthesized by Astellas, compound 35c, was shown to be more potent in inhibiting recombinant rat (rrVAP-1) than rhVAP-1 with IC_50_ values of 13 and 230 nM, respectively. When administered subcutaneously to STZ-induced diabetic rats, compound 35c significantly reduced ocular permeability [24]. The fluoroallylamine compound originally synthesized by Pharmaxis, PXS-4728A, demonstrated that selective VAP-1 inhibition reduced leukocyte adhesion and migration to sites of lung inflammation in various rat and mouse disease models with nearly equipotent inhibition of rhVAP-1 and rodent fat tissue homogenates [25].

The crystal structure of VAP-1 revealed that VAP-1 is a type 2 transmembrane protein, consisting of two monomers. The extracellular region has 3 domains (D2, D3, and D4), with residues from each of these domains composing the active site, but a majority are from the D4 domain [18, 20]. In addition to the enzymatic site, there are 3 other motifs that regulate leukocyte adhesion—the RGD motif, sites of sialic acid modification, and adhesion epitopes [26]. A homology model study comparing mouse, rat, monkey, and human VAP-1 revealed that rodent VAP-1 has a narrower and more hydrophilic active site channel than primate VAP-1, suggesting that rodent VAP-1 would favor smaller hydrophilic substrates/inhibitors [20]. As rodents are a common model organism for evaluating inhibitor efficacy, it is important to appreciate that difference in the binding efficiency across species.

In the present study, we have expanded on the comparisons of rodent VAP-1 to human VAP-1 using recombinant mouse VAP-1 (rmVAP-1), recombinant rat VAP-1 (rrVAP-1), and recombinant human VAP-1 (rhVAP-1) to determine the *in vitro* oxidative deamination activity of the most common marker substrate, BA, as well as the biogenic amines: tryptamine and 2-phenylethylamine. Furthermore, we have characterized the inhibitor potency of several well-characterized VAP-1 inhibitors against each of these 3 recombinant proteins.

## 2. Materials and Methods

### 2.1. Recombinant Enzymes

Recombinant human VAP-1 (rhVAP-1; catalog # 3957-AO-010) and mouse VAP-1 (rhVAP-1; catalog # 6107-AO-010) were purchased from R&D Systems (MN), while recombinant rat VAP-1 (rrVAP-1) was a custom product produced at R&D Systems.

### 2.2. Reagents

Semicarbazide and hydralazine were purchased from Sigma. PXS-4728A (now known as BI 1467335) was purchased from Axon MedChem. Compound 35c was synthesized as described by Inoue et al. [19] while LJP-1207 was synthesized as described by Salter-Cid et al. [22].

### 2.3. VAP-1 Oxidative Deamination Assay

The enzymatic activity of recombinant VAP-1 and inhibitor potency was determined using a variation of the fluorometric assay for measuring hydrogen peroxide formation [22]. Briefly, the assay measures the H_2_O_2_ generated during the oxidative deamination of an amine substrate (BA for inhibition purposes) by converting Amplex® Red to the fluorescent product, resorufin, via horseradish peroxidase (HRP). The assay was optimized for time, protein, and reagent contents and performed in a 96-well plate with a physiologically relevant reaction buffer (10 mM NaHCO_3_, pH 7.4) that has been shown to accentuate amine oxidase activity [27]. Stock solutions of Amplex Red (1 mM), HRP (10 U/mL), the substrates (200 *μ*M BA for inhibition), and inhibitor were prepared in reaction buffer and added to the appropriate wells, that the final concentration in each well was 0.05 mM Amplex Red, 0.5 U/mL HRP, and 50 *μ*M BA (saturating condition) in a total reaction volume of 0.1 mL. Inhibitors were diluted in DMSO to 20x and distributed to the appropriate wells including no inhibitor and each plate contained 1 *μ*M LJP-1207 as a positive control in duplicate. Each inhibitor concentration had duplicate wells without BA to determine the deamination activity that the inhibitor contributed to the H_2_O_2_ generated as background controls.

Standard curves were prepared for each plate by serially diluting H_2_O_2_ in reaction buffer with equivalent concentrations of Amplex Red and HRP. A 10 *μ*M solution of resorufin was prepared in reaction buffer and was dispensed in duplicate as the positive control for maximum fluorescence. Plates were incubated for 30 minutes at 37°C, protected from light under foil to minimize the resorufin artifactually formed when Amplex Red is exposed to light [28]. The plate was read at 530/35 emission and 590/35 excitation. The mean fluorescence of the background was subtracted from the mean fluorescence of the inhibitor concentration in the presence of BA. The percent control was calculated using equation (1). The percent inhibition was calculated using equation (2).

(1) $$\begin{array}{c} \%\text{control}=\left(\frac{\text{average fluorescence with inhibitor}-\text{background}}{\text{average fluorescence of reaction without inhibitor}-\text{background}}\right)\times 100, \end{array}$$

(2) $$\begin{array}{c} \%\text{inhibition}=100-\%\text{control}. \end{array}$$

Data processing was performed using Prism software. A two-way ANOVA was used to compare the mean IC_50_ values among the three species sources of recombinant VAP-1 using Bonferroni's test for multiple comparisons.

## 3. Results

**Figure 1:**
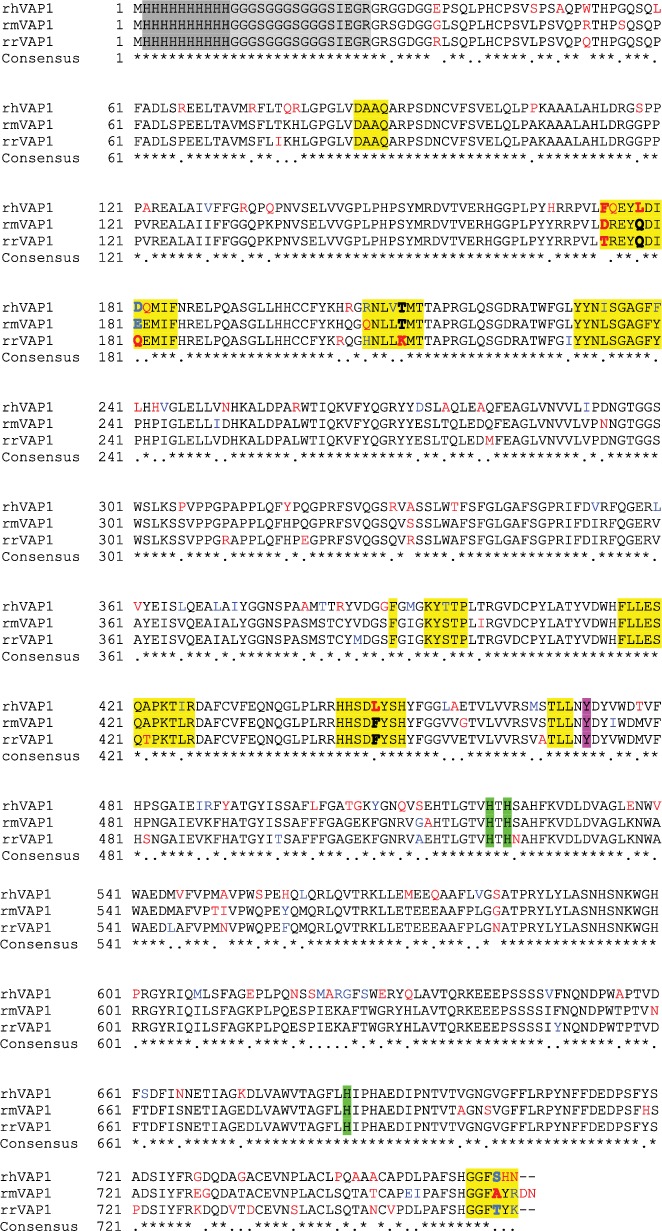
Sequence alignment of recombinant mouse VAP-1 (rmVAP-1), recombinant rat VAP-1 (rrVAP-1), and recombinant human VAP-1 (rhVAP-1). Dark grey highlight indicates the HIS tag. Light grey tag indicates the linker sequence. Amino acids that reside in the active site pocket are highlighted yellow. The amino acids that vary between species are in red or blue. The blue color indicates that the amino acids have side chains with similar properties (nonpolar, polar, acidic, or basic). The amino acids that are bolded are believed to be key for defining the properties of the active site channel. The tyrosine that is converted into topaquinone is highlighted in magenta. The histidine residues that coordinate the copper are highlighted in green.

Recombinant proteins were produced using *Spodoptera frugiperda*, Sf21 (baculovirus) system at R&D Systems. All constructs consisted of an N-terminal methionine, 10x-His tag, linker (GGGSGGGSGGGSIEGR), and amino acid 27 to the final amino acid. The sequences of the recombinant VAP-1 from human, mouse, and rat gene sequences used within this study are depicted in Figure 1 where the amino acids that vary between species are in red or blue. The blue color indicates amino acids that have side chains with similar properties (nonpolar, polar, acidic, or basic), whereas residues colored red differ between species and do not have side chains with similar properties. The amino acids highlighted in yellow comprises the active site, and those amino acids which are bolded are believed to be key for defining the significant differences between rodent and human active site channels. The tyrosine that is posttranslationally converted into topaquinone is highlighted in magenta. The histidine residues that coordinate the copper ion are highlighted in green. The overall sequences of mouse and rat VAP-1 are 83% and 80% identical, respectively, to human VAP-1. The active site is highly conserved, with 85% and 75% homology to humans for mice and rats, respectively [20, 29, 30].

Molecular modeling has predicted that rodent VAP-1 has a narrower and more hydrophilic active site channel than human VAP-1. These same authors compared three novel pyridazinones in rhVAP-1 and rmVAP-1, demonstrating that they were potent human VAP-1 inhibitors but were poor inhibitors of the mouse homolog. They concluded *in vivo* preclinical models other than rodents should be considered for testing efficacy (e.g., transgenic mice expressing hVAP-1) [18]. However, other researchers have successfully used rat and mouse models of inflammation to demonstrate the anti-inflammatory effects of VAP-1 inhibition, without expressing human VAP-1 [10–14]. Henceforth, we have compared *in vitro* VAP-1 inhibition by a variety of compounds in purified recombinant human, rat, and mouse VAP-1 using an optimized inhibition screening assay that reports the % inhibition while also accounting for inhibitor turnover as a substrate.

These inhibitors tested represent different functional groups that interact with active site and various molecular sizes with the upper three compounds in Table 1 being well-studied hydrazines: semicarbazide (SEM), hydralazine (HYD), and LJP-1207. In addition, the proprietary compounds from Astellas compound 35c (a guanadine) and Boehringer Ingelheim PXS-4728A (a fluoroallylamine) were tested. The IC_50_ values (mean ± standard deviation) of these inhibitors are shown in Table 1. Statistical analysis of the IC_50_ values is indicated in Table 1. Semicarbazide was the least potent of the five inhibitors tested and with nearly a 10-fold difference in IC_50_ between human and rat VAP-1 (85.9 *μ*M vs. 993 *μ*M) with the mouse VAP-1 IC_50_ value (295 *μ*M) in between. PXS-4728A was the most potent inhibitor under our assay conditions showing little difference between mice, rats, and humans with IC_50_ values of 33.8, 20.6, and 15.0 nM, respectively. These IC_50_ values for PXS-4728A were slightly higher, but in close agreement with those previously published in mouse, rat, and human VAP-1 of 21, 18 and 5 nM, respectively, albeit not all from recombinant sources [25]. LJP-1207 was also fairly potent with minor differences between mice, rats, and humans with IC_50_ values of 102, 120, and 252 nM, respectively. The reported IC_50_ for LJP-1207 was 7.5 nM for rats and 17 nM for humans, but again, these values were determined using VAP-1 from either rat lung or human umbilical cord homogenates and a radiolabeled substrate [22]. Compound 35c exhibited submicromolar IC_50_ values and was most potent against human VAP-1 (22.5 nM) and least potent against mouse VAP-1 (414 nM), while previously reported IC_50_ for compound 35c was 20 nM against human VAP-1 or 72 nM against rat VAP-1; these values were determined using a recombinant enzyme suspension prepared from Chinese hamster ovary cells stably expressing a human or rat VAP-1 and ^14^C-BA [19]. Hydralazine (HYD) had single digit micromolar IC_50_ values under these assay conditions yet was most potent in mice (1.18 *μ*M) and least potent in humans (7.75 *μ*M). The IC_50_ of HYD in rat VAP-1 was 3.13 *μ*M but was 1000x higher than previously reported IC_50_ of 25 nM determined using VAP-1 from rat aorta homogenates and ^14^C-BA [31]; however, the IC_50_ of HYD in human VAP-1 from aorta membrane preparations and recombinant enzymes using an LC-MS/MS method to detect benzaldehyde formation were 6.40 and 26.5 *μ*M, respectively [32]. These values were in close agreement with the 7.75 *μ*M value reported here.

**Figure 2:**
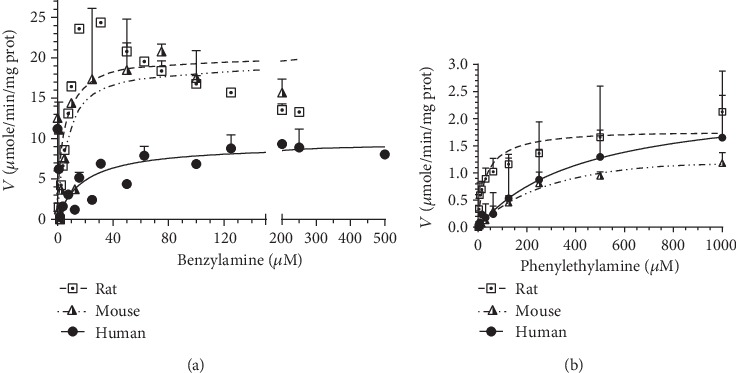
Substrate saturation curves for (a) benzylamine and (b) phenylethylamine in recombinant mouse VAP-1 (half-filled triangle with hatched line), recombinant rat VAP-1 (boxed dot with dashed line), and recombinant human VAP-1 (filled circles with solid lines). Lines represent nonlinear fit of three separate experiments run at varying concentrations in triplicate.

Substrate saturation curves for BA and PEA in recombinant human, rat, and mouse VAP-1 were also evaluated using the optimized fluorometric assay detecting hydrogen peroxide production as depicted in Figure 2 with the *V*_max_ and *K*_M_ values listed in Table 2. Both substrates were incubated with recombinant VAP-1 in triplicate on three separate plates. The maximal reaction rate (*V*_max_) for BA between the three species was similar ranging from 11.2 to 20.2 *μ*moles/min/mg protein, with rodent enzymes having slightly higher rates than the human VAP-1. The *K*_M_ for BA did vary with values of 13.1, 4.38, and 45.3 *μ*M in mouse, rat, and human VAP-1. The *V*_max_ for PEA between the three species was again similar ranging from 1.49 to 7.15 *μ*moles/min/mg protein, but in this case, the rodent enzymes had slightly lower rates than the human VAP-1. The *K*_M_ for PEA varied substantially with values of 263, 35.9, and 5510 *μ*M in mouse, rat, and human VAP-1. Tyramine and tryptamine were also explored as substrates for VAP-1 in each of the three species (*n* = 1 plate), with *K*_M_ values for TYR of 8.31 and 299 *μ*M and *V*_max_ values of 1.19 and 0.40 *μ*moles/min/mg protein in rat and human VAP-1, respectively. Values were not obtained for TYR in mouse VAP-1 nor tryptamine in any of the species due to such low reaction rates. The substrate potential of the endogenous aromatic amine tyramine, phenylethylamine (PEA), dopamine, histamine, and tryptamine has been compared to BA in *in vitro* tissue preparations with *K*_M_ values ranging from 2.8 to 6.2 *μ*M for BA, 10 to 44 *μ*M for PEA, and 40 to 52 *μ*M for TYR in rats and 110 to 116 *μ*M for BA, 7.90 mM for PEA, and 10.5 mM for TYR in humans [33]. These comparative values are in similar ranges to the data presented for the recombinant proteins and demonstrate that rodent VAP-1 has a slightly higher affinity for BA and PEA than human VAP-1 as evidenced by their lower *K*_M_ values. The rodent active site was predicted to favor small hydrophilic substrates/inhibitors whereas the human active sight would favor larger hydrophobic substrates [20]. The lower *K*_M_ values for BA and PEA in rodent vs. human enzymes support this hypothesis.

## 4. Discussion

VAP-1 deaminates primary amines as part of its mechanism for leukocyte adhesion to vascular tissues [3]. VAP-1 activity and protein levels increase during inflammation [10]. VAP-1 inhibition curtails leukocyte recruitment to sites of inflammation in vivo [15] as well as the transendothelial migration of leukocytes in vitro [15, 16]. Since VAP-1 has both enzymatic activity and inflammatory properties, it has become a promising therapeutic target.

Species differences in VAP-1 inhibitor sensitivity were previously documented using VAP-1 activity in plasma or homogenized tissues [33]. Homology models were used to predict species-specific ligand specificity [18, 20]. The data presented here compares the species-specific inhibition and substrate selectivity of purified recombinant mouse, rat, and human VAP-1 in an optimized in vitro assay. We investigated a diverse class of small-molecule VAP-1 inhibitors, which all had primary amine moieties in common.

Although VAP-1 is also known as SSAO (semicarbazide-sensitive amine oxidase), we demonstrated that VAP-1 is not very sensitive to semicarbazide inhibition. The semicarbazide IC_50_ values are 10–10000 higher than the other four compounds tested. This finding is corroborated by a cell-based inhibition assay of VAP-1, where 100x more semicarbazide was required than LJP-1207 (another hydrazine) for inhibition [17]. Additionally, semicarbazide is not specific to VAP-1; it also inhibits lysyl oxidase (LOX) [34–36].

Hydralazine is a vasodilator prescribed to treat high blood pressure. Like semicarbazide, hydralazine contains a hydrazine moiety. Hydralazine is a potent, irreversible inhibitor of VAP-1; this inhibition likely contributes to its mechanism for vasodilation [37, 38]. Since semicarbazide, LJP-1207, and hydralazine are all irreversible inhibitors of VAP-1, the IC_50_ value has a different inference when comparing noncovalent reversible inhibitors due to the time-dependent nature of the reaction instead of competitive equilibrium [39]. However, the coincubation time of inhibitors and the substrate benzylamine for 30 minutes in the assay presented here should allow for ample time for covalent inhibition.

The results presented here show that these smaller hydrazine inhibitors demonstrated some species differences beyond just humans compared to rodents. Human VAP-1 was more sensitive (lowest IC_50_ concentration) to semicarbazide but was least sensitive to hydralazine and LJP-1207. Although hydralazine had a lower IC_50_ in rats compared to humans, this was not significant due to the larger error in the human data. However, the IC_50_ of hydralazine was significantly higher in rat compared to mouse VAP-1.

The larger hydrophobic compounds from Astellas (compound 35c; a guanidino compound) and Boehringer Ingelheim (PXS-4728A; an allyl halide) are predicted to bind noncovalently in the channel leading to the active site. The channel in human VAP-1 is larger and more hydrophobic than that in rodent VAP-1. It was therefore predicted that the larger more hydrophobic inhibitors would have higher binding affinity for human VAP-1 compared to rodent VAP-1 [18, 20]. Although the sensitivity of these two inhibitors was the lowest in the mouse enzyme, we found no significant differences between mouse, rat, and human VAP-1.

There are several instances where the sequence is more homologous between rats and humans than mice and rats; of particular interest may be the amino acid 761 in the human sequence (762 in Figure 2), which is a serine in humans, threonine in rats, and alanine in humans. This amino acid is in the putative active binding site. The serine and threonine are large and polar, whereas the alanine is small and nonpolar. In general, the smaller hydrazines (LJP-1207 and hydralazine) had less sensitivity for the human VAP-1 than for the rat and mouse enzymes.

The physiological substrates of VAP-1 are not well established across species, but benzylamine (BA) has long been used as a substrate for oxidative deamination assays for function and was first shown to have the highest rate of oxidation in horse, pig, and dog serum while being sensitive to carbonyl inhibitors [40]. The comparative findings detailed here support that BA is the most appropriate substrate for measuring in vitro VAP-1 activity in rodents and humans. Tyramine and 2-phenylethylamine (PEA) are a trace amine found in the central nervous system due to their rapid deamination [41, 42]. It is believed that monoamine oxidase B is the primary amine oxidase responsible for this deamination [43, 44], but others have shown that VAP-1 is involved in deaminating PEA, TYR, and tryptamine in rat and human tissues in vitro [33]. We have corroborated that PEA and TYR are also deaminated by purified recombinant rat and human VAP-1, with rat VAP-1 having the highest affinity for BA, PEA, and TYR. Mouse VAP-1 had intermediate affinity for BA and PEA, but TYR was not a substrate for mouse VAP-1 under these assay conditions.

## 5. Conclusion

In conclusion, we have demonstrated differences in inhibitor sensitivity and substrate affinity for purified recombinant VAP-1 from humans, rats, and mice. The data presented here confirm the findings of a previous manuscript comparing inhibitor sensitivity in mouse and human VAP-1 [18]; however, our findings show that inhibitors affect rat VAP-1 in a manner that is not always more similar to mouse VAP-1 than human VAP-1. The IC_50_ of LJP-1207 in rats and mice were similar and significantly lower from that in humans, whereas the IC_50_ of hydralazine was significantly higher in rat compared to mouse VAP-1, but not significantly different from human VAP-1. These species differences highlight the opportunity to compare in vitro VAP-1 inhibition prior to advancing the inhibitors to in vivo animal models in order to select the most appropriate rodent species.

## Figures and Tables

**Table 1:** Summary of inhibition in recombinant VAP-1 from mice, rats, and humans. IC_50_ values are the mean and standard deviation of three separate determinations by nonlinear fit at varying concentrations in triplicate. Significance between mice and rats is indicated by ŧ. Significance between humans and mice is indicated by @. Significance between humans and rodents is indicated by ∗.

Compound name	Chemical structure	IC_50_mean ± SD (*μ*M)					
		Determined as described in Materials and Methods			Previously reported		
		Mouse	Rat	Human	Mouse	Rat	Human
Semiarid (SEM)	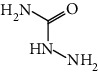	295 ± 29.0	993 ± 1215	85.9 ± 12.9^@^	NR	NR	NR

Hydralazine (HYD)	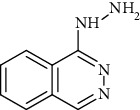	1.18 ± 0.092^ŧ^	3.13 ± 0.123	7.75 ± 2.32	NR	0.025 [31]	6.4 26.5 [32]

LJP-1207	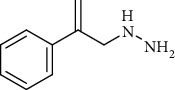	0.102 ± 0.0478	0.120 ± 0.0067	0.252 ± 0.0225^∗^	NR	0.0075 [22]	0.017 [22]

Astellas compound 35c	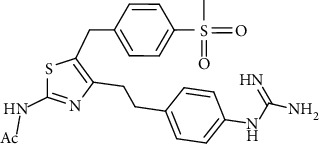	0.414 ± 0.113	0.0504 ± 0.0068	0.0225 ± 0.0069	NR	0.072 [19]	0.02 [19]

PXS-4728A	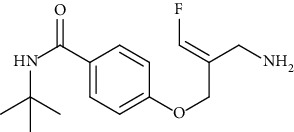	0.0338 ± 0.0098	0.0206 ± 0.0023	0.015 ± 0.006	0.021 [25]	0.018 [25]	0.005 [25]

^ŧ^ *p* < 0.05 mouse vs. rat; ^∗^*p* < 0.05 human vs. mouse and rat; ^@^*p* < 0.05 human vs. mouse. NR: not reported.

**Table 2:** Michaelis-Menten parameters for benzylamine (BA) and phenylethylamine (PEA) in recombinant mouse, rat, and human VAP-1. *K*_M_ and *V*_max_ values are the mean and standard deviation of three separate determinations by nonlinear fit at varying concentrations in triplicate.

Recombinant VAP-1 species	Benzylamine (BA)		Phenylethylamine (PEA)	
	*K*_M_ (*μ*M)	*V*_max_ (*μ*moles/min/mg prot)	*K*_M_ (*μ*M)	*V*_max_ (*μ*moles/min/mg prot)
Mouse	13.1 ± 11.4	20.1 ± 3.38	263 ± 0.0640	1.49 ± 0.153
Rat	4.38 ± 0.361	20.2 ± 0.863	35.9 ± 0.0218	1.78 ± 0.0539
Human	45.3 ± 50.7	11.2 ± 3.22	5510 ± 5510	7.15 ± 4.21

## Data Availability

The IC_50_, substrate saturation curves, *K*_M_, and *V*_max_ data used to support the findings of this study are included within the article.
